# Reconsideration of In-Silico siRNA Design Based on Feature Selection: A Cross-Platform Data Integration Perspective

**DOI:** 10.1371/journal.pone.0037879

**Published:** 2012-05-24

**Authors:** Qi Liu, Han Zhou, Juan Cui, Zhiwei Cao, Ying Xu

**Affiliations:** 1 Department of Bioinformatics, Tongji University, Shanghai, China; 2 Computational Systems Biology Laboratory, Department of Biochemistry and Molecular Biology, and Institute of Bioinformatics, University of Georgia, Athens, Georgia, United States of America; 3 College of Computer Science and Technology, Jilin University, Changchun, China; University of Iowa, United States of America

## Abstract

RNA interference via exogenous short interference RNAs (siRNA) is increasingly more widely employed as a tool in gene function studies, drug target discovery and disease treatment. Currently there is a strong need for rational siRNA design to achieve more reliable and specific gene silencing; and to keep up with the increasing needs for a wider range of applications. While progress has been made in the ability to design siRNAs with specific targets, we are clearly at an infancy stage towards achieving rational design of siRNAs with high efficacy. Among the many obstacles to overcome, lack of general understanding of what sequence features of siRNAs may affect their silencing efficacy and of large-scale homogeneous data needed to carry out such association analyses represents two challenges. To address these issues, we investigated a feature-selection based in-silico siRNA design from a novel cross-platform data integration perspective. An integration analysis of 4,482 siRNAs from ten meta-datasets was conducted for ranking siRNA features, according to their possible importance to the silencing efficacy of siRNAs across heterogeneous data sources. Our ranking analysis revealed for the first time the most relevant features based on cross-platform experiments, which compares favorably with the traditional in-silico siRNA feature screening based on the small samples of individual platform data. We believe that our feature ranking analysis can offer more creditable suggestions to help improving the design of siRNA with specific silencing targets. Data and scripts are available at http://csbl.bmb.uga.edu/publications/materials/qiliu/siRNA.html.

## Introduction

RNA interference (RNAi) is a gene silencing phenomenon mediated by a short interfering RNA (siRNA) which comes from a double-stranded RNA (dsRNA). The RNAi process is to introduce a siRNA into the cytoplasm, where the guide strand of the siRNA is incorporated into the RNA-induced silencing complex (RISC). As RISC binds with the target mRNA, the guide strand of the siRNA pairs up with the complementary mRNA sequence, leading to post-transcriptional gene silencing [1], [2]. The silencing effect of RNAi on specific genes makes it a powerful tool in gene function studies, drug target discovery and disease treatment [3]–[7].

The efficacy of different siRNAs may vary widely due to the specific characteristics of the siRNA sequences [8]. Considerable efforts have been made to study the silencing effects of siRNAs, and a number of features have been previously identified, which may affect the efficacy of a siRNA such as GC content, position-dependent nucleotide composition and the symmetric 3′ TT overhangs [9]–[12]. More recent studies have proposed a number of new rules, derived by employing more sophisticated statistical and machine learning methods as well as based on improved understanding about the RNA silencing mechanism [11], [13]–[18]. However, these empirical rules were often not discriminative enough between highly efficacious and inefficacious siRNAs [19] when tested on independent data. A key issue is that the proposed rules were generally not derived from a comprehensive dataset that covers the silencing effects by different siRNAs, which has led to poor performance by the existing siRNA design tools as reported in the literature. For instance, Saetrom et al. claimed that the sequence information alone can determine the efficacy of siRNAs [10] while several other groups suggested that thermodynamic features are important to siRNAs effectiveness (a comprehensive list of different concerns on related features in siRNA design is provided in the RESULTS section).

A number of RNAi datasets are publicly available but each dataset was typically generated by a different group possibly using a different platform under specific experimental conditions, making integrated analysis and utilization of these datasets a challenge. For example, a variety of assays/platforms/scales were used when measuring and assessing the siRNA efficacy, such as different cell types (Hela, fibroblasts), test methods (Western Blotting, real-time PCR) or siRNA delivery methods (vectors method, synthetic oligos method). In addition, very different concentrations of siRNAs might have been used in different experiments. We observed from our own previous study that generally the siRNA efficacy for different platforms cannot be easily compared, hence making a simple integration of heterogeneous datasets hardly useful [20].

To address these issues, an effective integration strategy is needed, which should maximally utilize information from different datasets to provide a reliable association analysis between features and the siRNA efficacy than analyses based on any specific dataset as done in the previous studies. All these require us to re-consider the current in-silico siRNA design strategies and rectify several confounding and potentially conflicting viewpoints on specific features related to siRNA design.

In this study, the features important to siRNA designs across different datasets were identified, including compositional, thermodynamic and structural features of siRNAs. Joint feature ranking was achieved by integration of feature selections using three learning methods, namely the 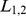-norm regularization, 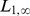-norm regularization and trace norm regularization [21], [22] on ten meta-datasets. Three ranked feature lists based on the three methods were integrated into one final ranking list. Our prediction results show an improved performance over the existing ones in terms of the silencing efficacy of the designed siRNAs.

## Methods

### Data sets

Ten siRNA efficacy datasets [17] were used in this study. The datasets were limited to siRNA sequences targeted at mammalian mRNAs. By convention, siRNA sequences were represented as anti-sense sequences from 5′ to 3′ and the siRNA potency was measured by the mRNA/protein product levels after gene silencing. Klingelhoefer et al. [17] noticed the heterogeneous nature of the ten datasets, and tried to combine the datasets into one through rescaling the data. However, this simple integration strategy has hardly led to satisfactory performance [20]. Nevertheless, the meta-datasets curated by Klingelhoefer et al. [17] provided a good starting point for us to carry out a comprehensive analysis on feature selection needed for siRNA design.

A detailed description of the ten datasets is presented in Table 1. Note that the siRecord data [23] was excluded from our study considering that the data used categorical values, unlike continuous values used in the other datasets when measuring the siRNA potency. The remaining datasets contain nearly all the RNAi data using numerical siRNA efficacy values reported so far. In total the datasets contain 4,482 unique and experimentally validated 19 nt siRNAs along with their efficacy values. All the datasets can be downloaded from the Supplementary Information.

**Table 1:** Data description for ten siRNA datasets.

ID	Dataset	Size	Source	siRNA sequence	Concentration
**1**	Novartis's data	2431	Huesken, et al., 2005	antisence	50 nM
**2**	Jagla's data	601	Jagla, et al., 2005	antisence	100 nM
**3**	Katoh's data	702	Katoh and Suzuki, 2007	sense	10/25 nM
**4**	Amgen-Dharmacon	239	Reynolds, et al., 2004	antisence	100 nM
**5**	Harborth'data	42	Harborth, et al., 2003	antisence	100 nM
**6**	Hsieh's data	108	Hsieh, et al., 2004	antisence	100 nM
**7**	Khvorova's data	10	Khvorova, et al., 2003	antisence	100 nM
**8**	Vickers'data	76	Vickers, et al., 2003	antisence	100 nM
**9**	Ui-Tei's data	50	Ui-Tei, et al., 2004	antisence	50 nM
**10**	Amarzguioui's data	223	Amarzguioui and Prydz, 2004	sense	25 nM

The same 497 features proposed by Klingelhoefer et al. [17] were adopted as the starting point of our study, including compositional, thermodynamic and structural features. The compositional features describe the occurrence of certain nucleotides at certain positions of the aligned siRNA sequences, including position-dependent nucleotide preference, GC content, presence of specific 2-, 3- and 4-mer sequence motifs, presence of the motifs that stimulate innate immune response and presence of palindromes. Thermodynamic features cover the binding free energies and stabilities of the folded structures; and structural features include secondary structure content. These features cover the vast majority of the features reported in recent studies, and provided a comprehensive starting feature set for our study. It should be noted that the features of the target mRNA do have important implications on siRNA potency [15], [24], however we did not take them into consideration in this study simply due to that we just keep the same feature set as proposed by Klingelhoefer et al. [17] for comparison purpose. The 497 features were listed in Table S1 for reference.

### Computational strategies for feature selection

**Figure 1:**
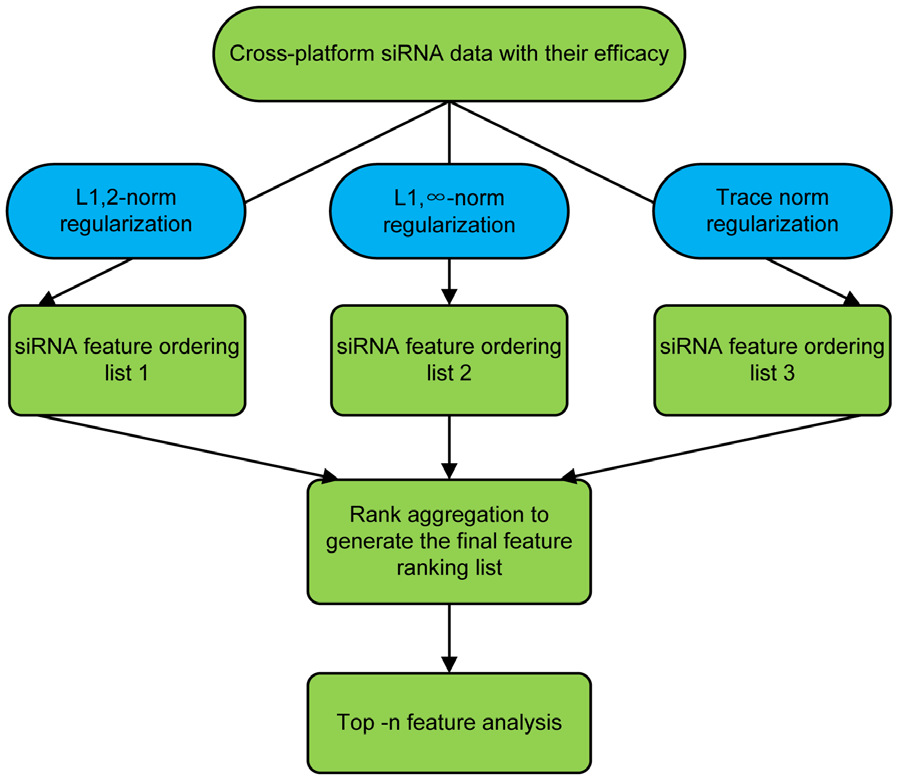
The computational framework for integrated cross-platform feature selection in siRNA design.

Data integration and feature selection were carried out by applying a linear regression model on data generated using three learning strategies. It exploits possible synergies across different datasets rather than combines them directly, to learn a predictor for siRNA efficacy. This predictor allows different regression tasks to enhance each other during the training process, which eventually makes the efficacy prediction and the selected features more reliable than when the datasets are used separately. Three regularization methods were used, namely, 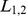-norm regularization, 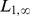-norm regularization and trace norm regularization [21], [22] to provide different constraints during model construction. The three methods will give rise to three different ranking lists of features for siRNA design, which will be then integrated to give one final rankling list. Figure 1 gives an outline of the whole procedure of our approach while the details of each step are given in the following subsections.

### Algorithms

#### Multi-Task Learning and Norm Regularization

For a given set of siRNAs represented as a set of feature vectors, traditional siRNA model is focused on a specific individual dataset to learn a regression model for the efficacy prediction. In our study, we aim at learning a joint efficacy prediction model for all the given datasets simultaneously, so a multi-task learning procedure is applied here. Under this framework, a “Task” represents an individual dataset used for the regression model for siRNA efficacy prediction. The goal of such multi-task learning model is to learn a set of sparse functions across all the tasks, by exploiting the possible synergies across different datasets rather than use only one dataset or combines them directly.

Specifically, the given datasets contain *N* tuples (the number of siRNA efficacy data is *N*), 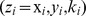 for 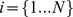, where 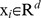 is a feature vector containing 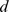 features for description for a specific siRNA, 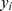
is the corresponding efficacy value, and 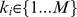 is the indicator specifying to which of the *M* tasks the example 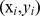
corresponds. The square loss: 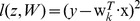
was adopted to learn the regression models, where 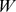 is a combination of the weight vector for each regression model which refers to 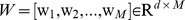 for *M* tasks and the 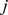-th row of 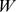 is denoted as 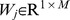, corresponding to the *i*-th feature in all *M* tasks. 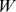 is enforced to be sparse to achieve the goal of cross-platform feature selection. In a sparse multi-task learning, joint sparsity across different tasks is obtained by adding the norm of the matrix *W* to the loss function, which leads to only a few non-zero rows of 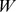, representing the leading features for cross-platform siRNA efficacy prediction. Overall, such a joint multi-task regression problem was formulated as the following optimization function to solve for 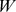:
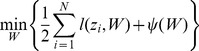
(1)where 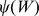
corresponds to the norm function. Note that the three different norm forms, i.e., 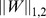 (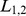-norm), 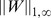 (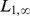-norm) and 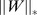 (Trace norm) are used respectively. The definition of each norm is defined as:
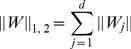
(2)

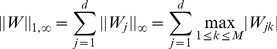
(3)

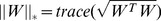
(4)The optimization problems resulting from the above sparse learning formulations were solved using the SLEP (Sparse Learning with Efficient Projections) package [22], by using Nesterov's method and the accelerated gradient method. Detailed information can be found in [22].

The reason for using three different norms rather than only one specific norm is explained below: at first, the common characteristic of all these regularized functions is that it encourages multiple tasks to share similar sparsity patterns. Basically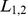-norm and 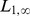-norm belong to the 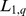-norm regularization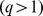, which contributes to representation sparsity, while trace-norm contributes to the matrix rank minimization. However, although each of the three norms can make sparse models in the siRNA efficacy regression, their induced levels of sparsity are unclear; and which norm should be applied for a specific case still remains an open problem [22]. For this particular reason, three different norms were adopted, and then integrated into one model to reveal the most un-biased features associated with the siRNA silencing efficacy, taking advantage of the integration of multi-view regularization norms.

#### Feature selection across multiple predictions

In sparse multi-task learning, the joint sparsity across different tasks is achieved by adding the regularization norm of matrix 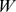 to the loss function, thus providing an efficient way to evaluate the joint feature importance in the siRNA design across multiple platforms. Based on the parameter 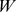 derived from the above three methods, we obtained 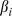 according to the following equation:
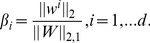
(5)If 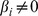, the 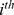
feature is the common feature; otherwise, the 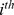 feature is not useful to the regression learning across different tasks, since its regression weights are zeros for all the tasks. The value of 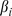 indicates the weight of the corresponding feature, providing a quantitative way to evaluate the importance of individual features for cross-platform siRNA design.

#### Rank integration

Rank integration was used to integrate the three feature lists derived based on three regularization forms [25]. An objective function is defined to cast the rank integration through solving an optimization problem:
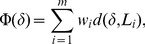
(6)where 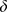 is an ordered list of length 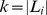, 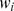 is the importance weight associated with list 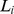, 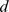 is a distance function that will be discussed below, and 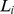 is the 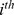 ordered list [26]–[28]. The intuition herein is to find a “super”-list that would be as “close” as possible to each individually ordered list simultaneously. In other words, 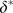 is calculated to minimize the total distance between 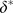 and 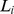
's, 
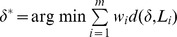
. The Kendall distance was selected here [25] to measure the distance between the ordered lists; and the Cross-Entropy Monte Carlo (CE) algorithm was applied to solve for the optimal objective value [25], [26].

#### T-test for feature significance evaluation

One-tailed, two samples, unequal variance t-tests were used to compare the mean activity for siRNAs which contain a given feature, with the mean activity of the remaining siRNA that are without such a feature, to generate the corresponding *p*-values for their significances. The threshold was set to be 0.05 as usual.

## Results

### Results of Ranking Integration

**Figure 2:**
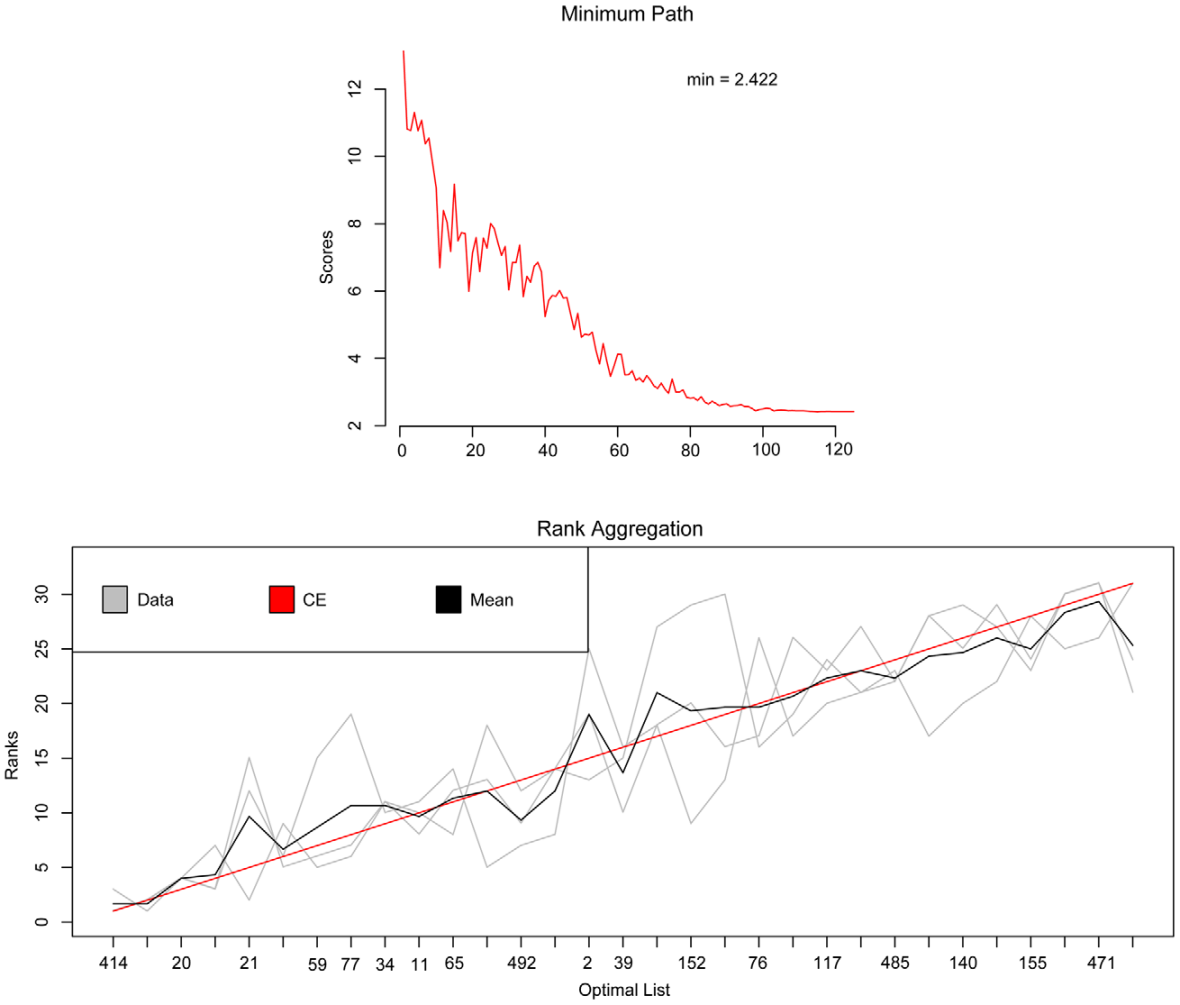
A representation of the integrated ranking results.

Our study is focused on an improved feature selection strategy for in-silico siRNA design based on cross-platform data integration. There are two main issues left to be addressed based on such an integrated study: (1) how to present an efficient data integration and feature selection model for siRNA design taking advantages of the largely distributed experimental siRNA data; and (2) how to rank these features to uncover their different contributions for screening highly efficient and specific siRNA as well as to rectify several confounding and conflicting viewpoints on specific features related to the current siRNA design. The computational procedure for addressing these problems is listed in Figure 1. Specifically, we ran our feature selection algorithm using three regression models. For each model, we used 10-fold cross-validation (CV) to conduct feature selection and test the prediction performance of the model. We selected the overlapping features across 10-fold CV to form a feature list in which features were ranked by their weight, and the predictive accuracy was evaluated by the average RMSEs shown in Table 2. It can be seen that the performance of the three models gave rise to nearly the same results, and the 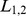-norm model is slightly superior to the other two. After the above procedure, we obtained three feature ranking lists from the regularization forms and the overlap 31 features were used to be aggregated. Rank integration was applied to obtain the final rank of the 31 features. Figure 2 shows a visual representation of the aggregation results. From the top plot, we can see that the performance stabilizes after roughly 100 iterations, implying the ranking result is convergent and will not change significantly anymore. In addition, the bottom plot clearly shows why the aggregation makes sense, on which the three light-gray lines represent the input lists, the red line represents the obtained solution to the optimization problem of ranking aggregation and the dark grey line represents the integrated optional average ranking list. Among them, the dark grey line and the red line fit quite well, indicating the CE algorithm successfully performs and achieve a satisfying result. It can also be seen that features ranked high in the final list usually belong to the lists as indicated by multiple intersecting lines.

**Table 2:** Accuracy of three regression models for siRNA efficacy prediction.

	RMSE									
Norm form	D1	D2	D3	D4	D5	D6	D7	D8	D9	D10
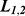 **-norm**	0.1521	0.2359	0.1493	0.2432	0.1062	0.2159	0.0155	0.1938	0.1921	0.2809
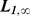 **-norm**	0.1502	0.2495	0.1584	0.2643	0.0864	0.2318	0.0000	0.2188	0.2893	0.2885
**Trace norm**	0.1575	0.2503	0.1624	0.2716	0.0813	0.2319	0.0018	0.2028	0.2690	0.2893

To identify whether the selected features are significantly preferred or avoided for efficient siRNAs, the correlation between the feature and the siRNA efficacy (product level variable) and *p*-values were calculated on dataset 1–4 and 10, respectively, as they have sufficient data to assure the sample sizes. Table 3 presents the correlation between the value of each feature and its siRNA efficacy, as well as the *p*-value for the significance of the feature. We also divided the features based on their feature categories (See Table S14 to Table S16) and rank them according to the correlation coefficients (*R*) respectively.

**Table 3:** Feature ranking and correlation coefficients for siRNA design derived from cross-platform data integration.

Rank ID	Feat No	Feature explanation	R	p-value	Support	Opposite
1	414	‘GG^a^in PS^b^[1,2]’	0.3716	0.0000	Lu and Mathews, 2008 [38];	Klingelhoefer et al., 2009[17];
					Matveeva et al., 2007 [14];	
					Shabalina et al., 2006 [13];	
2	40	‘U @ PS1’	0.2791	0.0003	Jagla et al., 2005 [29];	
					Katoh and Suzuki, 2007 [24];	
					Reynolds et al., 2004 [9];	
					Shabalina et al., 2006 [13];	
					Vert et al., 2006 [30];	
3	20	‘A @ PS19’	−0.1443	0.1572	Huesken et al.,2005 [11];	
					Matveeva et al., 2007 [14];	
					Shabalina et al., 2006 [13];	
4	431	‘GG in PS[18,19]’	−0.1684	0.1044	Klingelhoefer et al., 2009 [17];	
					Lu and Mathews, 2008 [38];	
					Matveeva et al., 2007 [14];	
					Shabalina et al., 2006 [13];	
5	21	‘G @ PS1’	−0.2137	0.0023	Matveeva et al., 2007 [14];	
					Shabalina et al., 2006 [13];	
6	494	‘GC content<0.55’	0.2441	0.0038	Matveeva et al., 2007 [14];	
					Chalk et al., 2004 [35];	
7	59	‘C @ PS1’	−0.1836	0.0075	Matveeva et al., 2007 [14];	
					Shabalina et al., 2006 [13];	
8	77	‘C @ PS19’	0.1120	0.2283	Huesken et al.,2005 [11];	
					Jagla et al., 2005 [29];	
					Katoh and Suzuki, 2007 [24];	
					Matveeva et al., 2007 [14];	
					Shabalina et al., 2006 [13];	
9	34	‘G @ PS14’	−0.1366	0.0876	Matveeva et al., 2007 [14];	
					Chalk et al., 2004 [35];	
10	11	‘A @ PS10’	0.1374	0.0288	Huesken et al.,2005 [11];	
					Jagla et al., 2005 [29];	
					Katoh and Suzuki, 2007 [24];	
					Matveeva et al., 2007 [14];	
					Reynolds et al., 2004 [9];	
					Vert et al., 2006 [30];	
11	65	‘C @ PS7’	−0.1264	0.0743	Katoh and Suzuki, 2007 [24];	
					Reynolds et al., 2004 [9];	
					Shabalina et al., 2006 [13];	
12	491	‘GC content<0.7’	0.2408	0.0024	Elbashir et al., [39];	
13	492	‘GC content<0.65’	0.2436	0.0043		
14	493	‘GC content<0.6’	0.2444	0.0170	Wang et al., 2004 [40];	
15	2	‘A @ PS1’	0.1269	0.1147	Jagla et al., 2005 [29];	
					Katoh and Suzuki, 2007 [24];	
					Matveeva et al., 2007 [14];	
					Reynolds et al., 2004 [9];	
					Shabalina et al., 2006 [13];	
16	39	‘G @ PS19’	0.0675	0.1340	Jagla et al., 2005 [29];	
					Katoh and Suzuki, 2007 [24];	
					Matveeva et al., 2007 [14];	
17	125	‘GCC in PS[1..19]’	−0.1272	0.2189	Klingelhoefer et al., 2009 [17];	
18	152	‘CUU in PS[1..19]’	0.1420	0.0673		Vert et al., 2006 [30];
19	92	‘CU in PS[1..19]’	0.1260	0.2946		
20	76	‘C @ PS18’	0.0705	0.2627	Shabalina et al., 2006 [13];	
21	157	‘CCC in PS[1..19]’	−0.0856	0.2423	Vert et al., 2006 [30];	
22	117	‘GGC in PS[1..19]’	−0.1259	0.1330		
23	33	‘G @ PS13’	−0.1174	0.1282	Matveeva et al., 2007 [14];	
24	485	‘GC content>0.45’	−0.1719	0.0388		
25	19	‘A @ PS18’	−0.0867	0.2145	Matveeva et al., 2007 [14];	
26	140	‘UCU in PS[1..19]’	0.1395	0.0562	Klingelhoefer et al., 2009 [17];	
27	49	‘U @ PS10’	0.0064	0.4615	Jagla et al., 2005 [29];	
					Katoh and Suzuki, 2007 [24];	
28	155	‘CCG in PS[1..19]’	−0.1352	0.1027	Vert et al., 2006 [30];	
29	115	‘GGG in PS[1..19]’	−0.1561	0.0092		
30	471	‘G stretch of length > = 3’	−0.1561	0.0092		
31	120	GUU in PS[1..19]’	0.0362	0.3726		Vert et al., 2006 [30];

a GG denotes the thermodynamic stability of dinucleotides in siRNA antisense strand.

b PS denotes the position of nucleotides in the siRNA sequence.

### Identified features

The following provides a discussion about the top features identified through the above analyses and a detailed comparison between our results with those from Klingelhoefer's et al. [17], which were considered representatives of the up-to-date large-scaled siRNA feature selection, aimed to provide some information regarding why they are relevant to siRNA design. It should be noted that the features discussed here are referred in Table 3 as rank IDs.

#### Compositional features

Nucleotide preferences or avoidance are identified at positions 1, 7, 10, 13–14 and 18–19.

Position 1: It was confirmed in our study that nucleotide U and A are preferable at position 1 (Rank ID: 2, 15) [9], [13], [14], [24], [29]. Similar with Klingelhoefer's results [17], our ranking list also indicates that U is more preferred than A at this position. Nucleotide G and C are depleted in the terminal position at the 5′ end of the antisense strand (Rank ID: 5, 7).

Position 7: nucleotide C at position 7 (Rank ID: 11) is associated with a negative effect on the siRNA efficacy, which is consistent with a few previous studies [9], [13], [24]. However, this feature was not found by Klingelhoefer et al [17].

Position 10: having an A at this position was positively correlated with the siRNA efficacy (Rank ID: 10). This has been suggested by numerous previous studies, including Klingelhoefer's et al. study [17], and is confirmed by our analysis; and our ranking also indicates that A has a more positive effect than U at this position (Rank ID: 27), which can be interpreted by the striking difference of correlation and *p*-value.

Positions 13 and 14: our analysis indicates that nucleotide G should be avoided at both positions (Rank ID: 23, 9). This is consistent with the observations made by Matveeva et al., Katoh et al. and Vert et al. [14], [24], [30], respectively. Klingelhoefer et al. [17] also reported the negative correlation between G14 and siRNA potency.

Position 18: In Klingelhoefer's et al results, nucleotide G at this position can reduce the siRNA efficacy [17]. However, our analysis shows that nucleotide A is found to be negatively correlated with the siRNA efficacy (Rank ID: 25), while nucleotide C at this position may increase the siRNA efficacy (Rank ID: 20). To identify the preference or avoidance at position 18, we carefully searched the previous literature and found our results have been proposed by Matveeva et al. and Shabalina et al., for A18 and C18 respectively.

Position 19: nucleotides C and G are preferable at this position (Rank ID: 8, 16). Meanwhile, A's undesirability (Rank ID: 3) is consistent with Klingelhoefer's et al suggestion [17].

The connection between the GC content and the siRNA efficacy is focused by a few research groups [9], [14], [31]–[34]. It has been argued that a high GC content may be a negative determinant of the functionality of a siRNA, inhibiting the dissociation of the duplex, which is necessary for RISC loading. Several published studies indicated that a very low GC content is also associated with the decreased functionality, presumably due to the lowered target affinity and specificity of the siRNA [33], [35]. However, how to quantitatively describe such association between the GC content and the siRNA functionality remains unclear. To address this issue, we investigated five GC content thresholds potentially important to siRNA efficacy, i.e., GC content<0.55, GC content<0.7, GC content<0.65, GC content<0.6 and GC content>0.45 (Rank ID: 6, 12, 13, 14, 24). As ‘GC content<0.55’ ranks much higher than the other three upper limits, we infer the proper upper-bound of GC content to be around 0.55. It is somewhat surprising that the lower bound has a negative correlation, which may be due to the inefficient siRNAs with high GC content (higher than 0.55). To confirm the speculation and identify the lower limit of GC content, we discard the siRNA data which have a GC content higher than 0.55 and selected those siRNAs with GC contents in the range of 45–55%, 40–55%, …, 5–55% with setting the GC content at an interval of 0.5 (the lower/higher thresholds are between 5–45%/55–95%, details showed in Supplementary Material). Statistical analysis indicated that when the lower limit is 25%, 51.7% of the siRNAs with GC content in the range of 25–55% are potent for a product level threshold of 0.3, which is the largest percentage among the candidate lower limits. Our analysis therefore infers that siRNA sequences with GC content in the range of 25–55% have an increased potency. This is similar with the range of GC content proposed by Reynolds et al. and Matveeva et al. [14] but differs from the GC content windows come from Klingelhoefer's et al. [17]. We also found that when the GC contents are in the range of 35–70% and 35–75% similar to the GC content 35–73% reported by Klingelhoefer's et al. [17], only 43.5% and 42.2% of the siRNAs are potent respectively.

#### Motif features

Four motifs: ‘CUU’ (Rank ID: 18), ‘CU’ (Rank ID: 19), ‘UCU’ (Rank ID: 26), ‘GUU’ (Rank ID: 31), are found to increase the siRNA efficacy. It is surprising that a new motif ‘UCU’ was identified, which has escaped the previous studies until its detection by Klingelhoefer et al. [17]. Furthermore, our analysis indicates that ‘UCU’ has a positive correlation with the siRNA efficacy, which is in accordance with the result of Klingelhoefer et al. [17]. Among the 4,482 siRNA sequences in our datasets, ‘UCU’ was found to occur in 1,345 sequences and 53% of these siRNAs were potent with product levels <0.3. We also made the detailed breakdown of the motif's occurrences by position in the siRNA sequence, which can be found in Tables S2to Table S13.

When looking for position-specific effects of ‘UCU’, they revealed that siRNAs with high potency always contain the ‘UCU’ motif at either end of the anti-sense sequence, which is accordant with Klingelhoefer's finding [17]. However, we observed a remarkable frequency drop for siRNAs with the motif at positions 6–8 and position 10–12 (Table S2) while Klingelhoefer's et al. [17] observed the drop at position 10–12 and position 11–13. We think our observation is more convincing since when analyzing the motif's occurrences at each position, we not only took into account the percentage of a certain motif at a specific position of potent siRNAs against of all siRNAs, but also considered the different percentage of a certain motif in different positions of potent siRNAs. From our point of view, the motif frequency drop at position 6–8 suggests the avoidance of ‘C’ at position 7 and the drop at position 10–12 may demonstrate that potent siRNAs prefer an ‘A’ at position 10, the cleavage site.

Therefore it can be noted that in our study, the positive correlation found between ‘UCU’ and silencing efficacy is likely to reflect a compositional characteristic of the sequences containing the specific motif. This is partly inconsistent with the conclusion of Klingelhoefer et al. [17].

Motif ‘CCG’, which was selected by our model, seems to relate with the motif ‘UCCG’ reported to increase siRNA efficacy by Klingelhoefer et al. [17] since both motifs contain ‘CCG’ but with opposite effect on siRNA efficacy. Of 820 siRNAs containing motif ‘CCG’, only 239 appear to be potent. Nevertheless, the position-specific analysis shows that when ‘CCG’ occurs at position 4–6, 14–16 and 16–18, it leads an increase of siRNA efficacy (Table S5). We also conduct a position-specific analysis on motif ‘UCCG’ to find some relations between ‘UCCG’ and ‘CCG’. Interestingly, we found that potent siRNAs share an enrichment of motif ‘UCCG’ at position 3–6, 7–10 and 15–18 (Table S7), which can explain the occurrence of ‘CCG’ at position 4–6 and 16–18 and indicates a ‘U’ in position 7.

Five motifs: ‘GCC’ (Rank ID: 17), ‘CCC’ (Rank ID: 21), ‘GGC’ (Rank ID: 22), ‘CCG’ (Rank ID: 28) and ‘GGG’ (Rank ID: 29) are found to be negatively correlated with the siRNA potency, among which ‘GCC’ has also been pointed out by Klingelhoefer et al. [17] while ‘CUU’ and ‘GUU’ were contrary to the results of Vert et al. [30]. It can be seen from Table 3 that motif ‘CUU’ has a remarkable positive correlation coefficient with p-value <0.1, tends to be positively affect siRNA efficacy. In the later position-specific analysis, we found a sharply frequency drop of motif ‘CUU’ at position 7–10 in potent siRNAs, indicating the avoidance of ‘C’ at position 7 once more (Table S8). We inferred that the contradiction between our result and that of Vert's et al. [30] may be caused by the difference of dataset. In Vert's study [30], they only used Novartis's data to select the features and inevitably missed some important information from other datasets.

Unlike motif ‘CUU’, although motif ‘GUU’ is represented in our feature list, this feature was not found to be significantly correlated with siRNA efficiency. The three 4-mer motifs selected by Klingelhoefer et al. [17] were not selected by our algorithm as well, which may due to comparative lack of data.In addition, motif ‘GGG’, along with the feature ‘G stretch of length > = 3’, suggest that continuous nucleotide ‘G’ should be removed in siRNA design, especially at position 1–3 and 17–19 after analyzing their position-specific effects (Table S13).

#### Thermodynamic features

Half of the top-4 features are thermodynamic features, indicating the critical role of the thermodynamic properties of a siRNA in duplex unwinding and strand retention by the complex [9], [36]. It is generally believed that siRNAs with high efficacy tend to have more instable 5′-ends in their antisense strands [9], [13]–[15]. Previous studies have found a strong correlation between the siRNA efficacy and the thermodynamic stability of dinucleotides (GG), especially for the first (GG1–2, Rank ID: 1) and the last (GG18–19, Rank ID: 4) dinucleotides on the siRNA antisense strand, which is consistent with our observation. Besides, GG1–4 and GG13–14 which were suggested by Klingelhoefer et al. [17] have not been selected by our models. Although GG13–14 was not in our result, two compositional features, nucleotide G in position13 and 14 also implied the positive effect of GG13–14 in potent siRNAs.

### Validation of selected features

We believed that in-vivo experimental studies on specific mutations in siRNAs will help to validate some of the identified features. Nevertheless, as a computational study, we can validate the selected features in-silico. In order to compare the features with other proposed ones, a linear ridge regression model was trained using 31 identified features on each of the 10 datasets for siRNA efficacy prediction to check the performance. We used 1,000 rounds of 2-fold cross-validation to train each model and test it. The average Root Mean Square Error (RMSE) was calculated for each experiment. Then linear regression models were constructed using 19 features reported by Klingelhoefer et al. [17] through 1,000 rounds of cross-validation, which will be served as the comparison. From Table 4, we can see that the prediction model with our feature set outperformed those from Klingelhoefer et al. [17] in 7 out of 10 datasets. For the smallest dataset (Dataset 7), model trained with Klingelhoefer's feature set [17] obviously gave rise to a better performance than that with our feature set. The poor performance of this experiment may result from the limited training samples, and such samples may lack of the most of the 31 selected features. When excluding the results of Dataset 7, the *p*-value for one-tailed pair t-test is 0.1, indicating that our prediction results are statistically significantly superior to those of Klingelhoefer's et al [17].

**Table 4:** Comparison between the model with 31 identified features (31_Feat) and the model with Klingelhoefer's et al. 19 features (19_Feat) for siRNA efficacy prediction.

	RMSE								
	D1	D2	D3	D4	D5	D6	D8	D9	D10
**31_Feat**	0.1557	0.2486	0.1615	0.2650	0.1592	0.2418	0.4474	0.2601	0.2205
**19_Feat**	0.1641	0.2516	0.1595	0.2662	0.1601	0.2517	0.4161	0.2546	0.2753

## Discussion

In this study, a joint feature selection across multiple siRNA-efficacy datasets was conducted. Three norm regularization methods were employed to exploit the feature space for siRNA design, which prevented the deviation resulted from using only one method, thus making the selected features more reliable and useful. The rank integration method was applied to obtain a more effective list of 31 features, which all have more significant correlations and p-values than the lower ranked ones.

We succeeded in confirming the majority of the previously reported features in silico, rectifying several conflicting rules as well as identifying several novel features.

Generally, siRNAs with high potency prefer to contain A or U towards the 5′ ends and G or C towards the 3′ ends of their guide strands. It should be noted that the p-values of some identified features, like G and C at the 3′ ends, are larger than 0.1. This can be explained by the fact that almost all the siRNAs are designed to have G or C at the 3′ ends on their antisense strand, no matter whether the siRNA is efficient or not. In the middle of the guide strands, the requirement of an A is related to the previously characterized A-cleavage site [37]. Nucleotide G depleted at positions 13 and 14 corresponds to the duplex stability in these two positions, suggesting a positive correlation between the free energy of the base pair in these positions and the siRNA efficiency of silencing.

Motif ‘UCU’ identified by our methods is essential for efficient RNA interference, which has been discovered as a novel feature by Klingelhoefer et al. [17]. Failing to identify the role of ‘UCU’ by previous studies may be caused by the insufficient siRNA efficacy data. Our analysis suggests that ‘UCU’ is a reliable feature positively correlated with the siRNA potency because it reflects and demonstrates the compositional characteristic in potent siRNAs.

By observing the general characteristics of the motifs that correlate with the increased or decreased potencies, we drew an opposite conclusion to that by Vert et al. [30], which is that potent siRNAs still can carry motifs containing C or G at 5′ in the antisense strand as long as they do not appear at specific positions, such as ‘G/C’ at 5′end and ‘C’ at position 7.

The finding that the dinucleotide thermodynamic stability at the 5′-end of the siRNA sequence, dG1–2, is more decisive for siRNA potency than the tetranucleotide stability, dG1–2 is also very interesting in terms of understanding the mechanism of siRNA incorporation into the RISC complex. The thermodynamic stability in the first two base pairs (GG1–2, GG18–19) is a good indicator of siRNA efficacy and thermodynamic consideration of four terminal nucleotides provides poorer correlation with siRNA efficacy which doesn't appear in our feature list. We infer that the thermodynamic stability of two base pairs at other positions may vary greatly even in potent siRNAs.

For the future research, we will include other features such as the properties of the target mRNAs as well as the matching properties between mRNAs and miRNAs. Considering the diversity and heterogeneity of data generated by different research groups, we strive to find novel methods to deal with the issue associated with the multiple platform data sources. A novel method named ‘heterogeneous transfer learning’ for utilizing siRNA datasets whose marginal distributions and output criteria are different is under preparation, which is expected to provide another new way to improve the reliability of the siRNA efficacy prediction from cross-platform data.

## Supporting Information

Table S1 **List of features employed in our study.**
(DOC)

Table S2 **Sequence-specific study of the impact of the motif ‘UCU’.**
(DOC)

Table S3 **Sequence-specific study of the impact of the motif ‘UCU’.**
(DOC)

Table S4 **Sequence-specific study of the impact of the motif ‘CCG’.**
(DOC)

Table S5 **Sequence-specific study of the impact of the motif ‘CCG’.**
(DOC)

Table S6 **Sequence-specific study of the impact of the motif ‘UCCG’.**
(DOC)

Table S7 **Sequence-specific study of the impact of the motif ‘UCCG’.**
(DOC)

Table S8 **Sequence-specific study of the impact of the motif ‘CUU’.**
(DOC)

Table S9 **Sequence-specific study of the impact of the motif ‘CUU’.**
(DOC)

Table S10 **Sequence-specific study of the impact of the motif ‘GUU’.**
(DOC)

Table S11 **Sequence-specific study of the impact of the motif ‘GUU’.**
(DOC)

Table S12 **Sequence-specific study of the impact of the motif ‘GGG’.**
(DOC)

Table S13 **Sequence-specific study of the impact of the motif ‘GGG’.**
(DOC)

Table S14 **Compositional feature ranking according to correlation coefficients (** ***R*** **).**
(DOC)

Table S15 **Motif feature ranking according to correlation coefficients (** ***R*** **).**
(DOC)

Table S16 **Thermodynamic feature ranking according to correlation coefficients (** ***R*** **).**
(DOC)
