# Supplementary material for: Reconsideration of In-Silico siRNA Design Based on Feature Selection: A Cross-Platform Data Integration Perspective
Source: PLoS One. 2012 May 24;7(5):e37879. doi: 10.1371/journal.pone.0037879 (PMC3360065; doi:10.1371/journal.pone.0037879)
Supplement: Table S1 — List of features employed in our study. (DOC) [file pone.0037879.s001.doc]

## Table S1. List of features employed in our study.

| **Feat.**  **No** | **Feat description** | **Feat.**  **No** | **Feat description** | **Feat.**  **No** | **Feat description** |
| --- | --- | --- | --- | --- | --- |
| **1** | 'GC content in PS^1)^[1..19]' | **167** | 'AAUG in PS[1..19]' | **333** | 'UUCC in PS[1..19]' |
| **2** | 'A @ PS1' | **168** | 'AAUU in PS[1..19]' | **334** | 'UCAA in PS[1..19]' |
| **3** | 'A @ PS2' | **169** | 'AAUC in PS[1..19]' | **335** | 'UCAG in PS[1..19]' |
| **4** | 'A @ PS3' | **170** | 'AACA in PS[1..19]' | **336** | 'UCAU in PS[1..19]' |
| **5** | 'A @ PS4' | **171** | 'AACG in PS[1..19]' | **337** | 'UCAC in PS[1..19]' |
| **6** | 'A @ PS5' | **172** | 'AACU in PS[1..19]' | **338** | 'UCGA in PS[1..19]' |
| **7** | 'A @ PS6' | **173** | 'AACC in PS[1..19]' | **339** | 'UCGG in PS[1..19]' |
| **8** | 'A @ PS7' | **174** | 'AGAA in PS[1..19]' | **340** | 'UCGU in PS[1..19]' |
| **9** | 'A @ PS8' | **175** | 'AGAG in PS[1..19]' | **341** | 'UCGC in PS[1..19]' |
| **10** | 'A @ PS9' | **176** | 'AGAU in PS[1..19]' | **342** | 'UCUA in PS[1..19]' |
| **11** | 'A @ PS10' | **177** | 'AGAC in PS[1..19]' | **343** | 'UCUG in PS[1..19]' |
| **12** | 'A @ PS11' | **178** | 'AGGA in PS[1..19]' | **344** | 'UCUU in PS[1..19]' |
| **13** | 'A @ PS12' | **179** | 'AGGG in PS[1..19]' | **345** | 'UCUC in PS[1..19]' |
| **14** | 'A @ PS13' | **180** | 'AGGU in PS[1..19]' | **346** | 'UCCA in PS[1..19]' |
| **15** | 'A @ PS14' | **181** | 'AGGC in PS[1..19]' | **347** | 'UCCG in PS[1..19]' |
| **16** | 'A @ PS15' | **182** | 'AGUA in PS[1..19]' | **348** | 'UCCU in PS[1..19]' |
| **17** | 'A @ PS16' | **183** | 'AGUG in PS[1..19]' | **349** | 'UCCC in PS[1..19]' |
| **18** | 'A @ PS17' | **184** | 'AGUU in PS[1..19]' | **350** | 'CAAA in PS[1..19]' |
| **19** | 'A @ PS18' | **185** | 'AGUC in PS[1..19]' | **351** | 'CAAG in PS[1..19]' |
| **20** | 'A @ PS19' | **186** | 'AGCA in PS[1..19]' | **352** | 'CAAU in PS[1..19]' |
| **21** | 'G @ PS1' | **187** | 'AGCG in PS[1..19]' | **353** | 'CAAC in PS[1..19]' |
| **22** | 'G @ PS2' | **188** | 'AGCU in PS[1..19]' | **354** | 'CAGA in PS[1..19]' |
| **23** | 'G @ PS3' | **189** | 'AGCC in PS[1..19]' | **355** | 'CAGG in PS[1..19]' |
| **24** | 'G @ PS4' | **190** | 'AUAA in PS[1..19]' | **356** | 'CAGU in PS[1..19]' |
| **25** | 'G @ PS5' | **191** | 'AUAG in PS[1..19]' | **357** | 'CAGC in PS[1..19]' |
| **26** | 'G @ PS6' | **192** | 'AUAU in PS[1..19]' | **358** | 'CAUA in PS[1..19]' |
| **27** | 'G @ PS7' | **193** | 'AUAC in PS[1..19]' | **359** | 'CAUG in PS[1..19]' |
| **28** | 'G @ PS8' | **194** | 'AUGA in PS[1..19]' | **360** | 'CAUU in PS[1..19]' |
| **29** | 'G @ PS9' | **195** | 'AUGG in PS[1..19]' | **361** | 'CAUC in PS[1..19]' |
| **30** | 'G @ PS10' | **196** | 'AUGU in PS[1..19]' | **362** | 'CACA in PS[1..19]' |
| **31** | 'G @ PS11' | **197** | 'AUGC in PS[1..19]' | **363** | 'CACG in PS[1..19]' |
| **32** | 'G @ PS12' | **198** | 'AUUA in PS[1..19]' | **364** | 'CACU in PS[1..19]' |
| **33** | 'G @ PS13' | **199** | 'AUUG in PS[1..19]' | **365** | 'CACC in PS[1..19]' |
| **34** | 'G @ PS14' | **200** | 'AUUU in PS[1..19]' | **366** | 'CGAA in PS[1..19]' |
| **35** | 'G @ PS15' | **201** | 'AUUC in PS[1..19]' | **367** | 'CGAG in PS[1..19]' |
| **36** | 'G @ PS16' | **202** | 'AUCA in PS[1..19]' | **368** | 'CGAU in PS[1..19]' |
| **37** | 'G @ PS17' | **203** | 'AUCG in PS[1..19]' | **369** | 'CGAC in PS[1..19]' |
| **38** | 'G @ PS18' | **204** | 'AUCU in PS[1..19]' | **370** | 'CGGA in PS[1..19]' |
| **39** | 'G @ PS19' | **205** | 'AUCC in PS[1..19]' | **371** | 'CGGG in PS[1..19]' |
| **40** | 'U @ PS1' | **206** | 'ACAA in PS[1..19]' | **372** | 'CGGU in PS[1..19]' |
| **41** | 'U @ PS2' | **207** | 'ACAG in PS[1..19]' | **373** | 'CGGC in PS[1..19]' |
| **42** | 'U @ PS3' | **208** | 'ACAU in PS[1..19]' | **374** | 'CGUA in PS[1..19]' |
| **43** | 'U @ PS4' | **209** | 'ACAC in PS[1..19]' | **375** | 'CGUG in PS[1..19]' |
| **44** | 'U @ PS5' | **210** | 'ACGA in PS[1..19]' | **376** | 'CGUU in PS[1..19]' |
| **45** | 'U @ PS6' | **211** | 'ACGG in PS[1..19]' | **377** | 'CGUC in PS[1..19]' |
| **46** | 'U @ PS7' | **212** | 'ACGU in PS[1..19]' | **378** | 'CGCA in PS[1..19]' |
| **47** | 'U @ PS8' | **213** | 'ACGC in PS[1..19]' | **379** | 'CGCG in PS[1..19]' |
| **48** | 'U @ PS9' | **214** | 'ACUA in PS[1..19]' | **380** | 'CGCU in PS[1..19]' |
| **49** | 'U @ PS10' | **215** | 'ACUG in PS[1..19]' | **381** | 'CGCC in PS[1..19]' |
| **50** | 'U @ PS11' | **216** | 'ACUU in PS[1..19]' | **382** | 'CUAA in PS[1..19]' |
| **51** | 'U @ PS12' | **217** | 'ACUC in PS[1..19]' | **383** | 'CUAG in PS[1..19]' |
| **52** | 'U @ PS13' | **218** | 'ACCA in PS[1..19]' | **384** | 'CUAU in PS[1..19]' |
| **53** | 'U @ PS14' | **219** | 'ACCG in PS[1..19]' | **385** | 'CUAC in PS[1..19]' |
| **54** | 'U @ PS15' | **220** | 'ACCU in PS[1..19]' | **386** | 'CUGA in PS[1..19]' |
| **55** | 'U @ PS16' | **221** | 'ACCC in PS[1..19]' | **387** | 'CUGG in PS[1..19]' |
| **56** | 'U @ PS17' | **222** | 'GAAA in PS[1..19]' | **388** | 'CUGU in PS[1..19]' |
| **57** | 'U @ PS18' | **223** | 'GAAG in PS[1..19]' | **389** | 'CUGC in PS[1..19]' |
| **58** | 'U @ PS19' | **224** | 'GAAU in PS[1..19]' | **390** | 'CUUA in PS[1..19]' |
| **59** | 'C @ PS1' | **225** | 'GAAC in PS[1..19]' | **391** | 'CUUG in PS[1..19]' |
| **60** | 'C @ PS2' | **226** | 'GAGA in PS[1..19]' | **392** | 'CUUU in PS[1..19]' |
| **61** | 'C @ PS3' | **227** | 'GAGG in PS[1..19]' | **393** | 'CUUC in PS[1..19]' |
| **62** | 'C @ PS4' | **228** | 'GAGU in PS[1..19]' | **394** | 'CUCA in PS[1..19]' |
| **63** | 'C @ PS5' | **229** | 'GAGC in PS[1..19]' | **395** | 'CUCG in PS[1..19]' |
| **64** | 'C @ PS6' | **230** | 'GAUA in PS[1..19]' | **396** | 'CUCU in PS[1..19]' |
| **65** | 'C @ PS7' | **231** | 'GAUG in PS[1..19]' | **397** | 'CUCC in PS[1..19]' |
| **66** | 'C @ PS8' | **232** | 'GAUU in PS[1..19]' | **398** | 'CCAA in PS[1..19]' |
| **67** | 'C @ PS9' | **233** | 'GAUC in PS[1..19]' | **399** | 'CCAG in PS[1..19]' |
| **68** | 'C @ PS10' | **234** | 'GACA in PS[1..19]' | **400** | 'CCAU in PS[1..19]' |
| **69** | 'C @ PS11' | **235** | 'GACG in PS[1..19]' | **401** | 'CCAC in PS[1..19]' |
| **70** | 'C @ PS12' | **236** | 'GACU in PS[1..19]' | **402** | 'CCGA in PS[1..19]' |
| **71** | 'C @ PS13' | **237** | 'GACC in PS[1..19]' | **403** | 'CCGG in PS[1..19]' |
| **72** | 'C @ PS14' | **238** | 'GGAA in PS[1..19]' | **404** | 'CCGU in PS[1..19]' |
| **73** | 'C @ PS15' | **239** | 'GGAG in PS[1..19]' | **405** | 'CCGC in PS[1..19]' |
| **74** | 'C @ PS16' | **240** | 'GGAU in PS[1..19]' | **406** | 'CCUA in PS[1..19]' |
| **75** | 'C @ PS17' | **241** | 'GGAC in PS[1..19]' | **407** | 'CCUG in PS[1..19]' |
| **76** | 'C @ PS18' | **242** | 'GGGA in PS[1..19]' | **408** | 'CCUU in PS[1..19]' |
| **77** | 'C @ PS19' | **243** | 'GGGG in PS[1..19]' | **409** | 'CCUC in PS[1..19]' |
| **78** | 'AA in PS[1..19]' | **244** | 'GGGU in PS[1..19]' | **410** | 'CCCA in PS[1..19]' |
| **79** | 'AG in PS[1..19]' | **245** | 'GGGC in PS[1..19]' | **411** | 'CCCG in PS[1..19]' |
| **80** | 'AU in PS[1..19]' | **246** | 'GGUA in PS[1..19]' | **412** | 'CCCU in PS[1..19]' |
| **81** | 'AC in PS[1..19]' | **247** | 'GGUG in PS[1..19]' | **413** | 'CCCC in PS[1..19]' |
| **82** | 'GA in PS[1..19]' | **248** | 'GGUU in PS[1..19]' | **414** | 'GG^2)^ in PS[1,2]' |
| **83** | 'GG in PS[1..19]' | **249** | 'GGUC in PS[1..19]' | **415** | 'GG in PS[2,3]' |
| **84** | 'GU in PS[1..19]' | **250** | 'GGCA in PS[1..19]' | **416** | 'GG in PS[3,4]' |
| **85** | 'GC in PS[1..19]' | **251** | 'GGCG in PS[1..19]' | **417** | 'GG in PS[4,5]' |
| **86** | 'UA in PS[1..19]' | **252** | 'GGCU in PS[1..19]' | **418** | 'GG in PS[5,6]' |
| **87** | 'UG in PS[1..19]' | **253** | 'GGCC in PS[1..19]' | **419** | 'GG in PS[6,7]' |
| **88** | 'UU in PS[1..19]' | **254** | 'GUAA in PS[1..19]' | **420** | 'GG in PS[7,8]' |
| **89** | 'UC in PS[1..19]' | **255** | 'GUAG in PS[1..19]' | **421** | 'GG in PS[8,9]' |
| **90** | 'CA in PS[1..19]' | **256** | 'GUAU in PS[1..19]' | **422** | 'GG in PS[9,10]' |
| **91** | 'CG in PS[1..19]' | **257** | 'GUAC in PS[1..19]' | **423** | 'GG in PS[10,11]' |
| **92** | 'CU in PS[1..19]' | **258** | 'GUGA in PS[1..19]' | **424** | 'GG in PS[11,12]' |
| **93** | 'CC in PS[1..19]' | **259** | 'GUGG in PS[1..19]' | **425** | 'GG in PS[12,13]' |
| **94** | 'AAA in PS[1..19]' | **260** | 'GUGU in PS[1..19]' | **426** | 'GG in PS[13,14]' |
| **95** | 'AAG in PS[1..19]' | **261** | 'GUGC in PS[1..19]' | **427** | 'GG in PS[14,15]' |
| **96** | 'AAU in PS[1..19]' | **262** | 'GUUA in PS[1..19]' | **428** | 'GG in PS[15,16]' |
| **97** | 'AAC in PS[1..19]' | **263** | 'GUUG in PS[1..19]' | **429** | 'GG in PS[16,17]' |
| **98** | 'AGA in PS[1..19]' | **264** | 'GUUU in PS[1..19]' | **430** | 'GG in PS[17,18]' |
| **99** | 'AGG in PS[1..19]' | **265** | 'GUUC in PS[1..19]' | **431** | 'GG in PS[18,19]' |
| **100** | 'AGU in PS[1..19]' | **266** | 'GUCA in PS[1..19]' | **432** | 'SUM of GG' |
| **101** | 'AGC in PS[1..19]' | **267** | 'GUCG in PS[1..19]' | **433** | 'GG in PS[1..4]' |
| **102** | 'AUA in PS[1..19]' | **268** | 'GUCU in PS[1..19]' | **434** | 'GG in PS[2..5]' |
| **103** | 'AUG in PS[1..19]' | **269** | 'GUCC in PS[1..19]' | **435** | 'GG in PS[3..6]' |
| **104** | 'AUU in PS[1..19]' | **270** | 'GCAA in PS[1..19]' | **436** | 'GG in PS[4..7]' |
| **105** | 'AUC in PS[1..19]' | **271** | 'GCAG in PS[1..19]' | **437** | 'GG in PS[5..8]' |
| **106** | 'ACA in PS[1..19]' | **272** | 'GCAU in PS[1..19]' | **438** | 'GG in PS[6..9]' |
| **107** | 'ACG in PS[1..19]' | **273** | 'GCAC in PS[1..19]' | **439** | 'GG in PS[7..10]' |
| **108** | 'ACU in PS[1..19]' | **274** | 'GCGA in PS[1..19]' | **440** | 'GG in PS[8..11]' |
| **109** | 'ACC in PS[1..19]' | **275** | 'GCGG in PS[1..19]' | **441** | 'GG in PS[9..12]' |
| **110** | 'GAA in PS[1..19]' | **276** | 'GCGU in PS[1..19]' | **442** | 'GG in PS[10..13]' |
| **111** | 'GAG in PS[1..19]' | **277** | 'GCGC in PS[1..19]' | **443** | 'GG in PS[11..14]' |
| **112** | 'GAU in PS[1..19]' | **278** | 'GCUA in PS[1..19]' | **444** | 'GG in PS[12..15]' |
| **113** | 'GAC in PS[1..19]' | **279** | 'GCUG in PS[1..19]' | **445** | 'GG in PS[13..16]' |
| **114** | 'GGA in PS[1..19]' | **280** | 'GCUU in PS[1..19]' | **446** | 'GG in PS[14..17]' |
| **115** | 'GGG in PS[1..19]' | **281** | 'GCUC in PS[1..19]' | **447** | 'GG in PS[15..18]' |
| **116** | 'GGU in PS[1..19]' | **282** | 'GCCA in PS[1..19]' | **448** | 'GG in PS[16..19]' |
| **117** | 'GGC in PS[1..19]' | **283** | 'GCCG in PS[1..19]' | **449** | 'SUM of GG4' |
| **118** | 'GUA in PS[1..19]' | **284** | 'GCCU in PS[1..19]' | **450** | 'Folding in PS[1..19]' |
| **119** | 'GUG in PS[1..19]' | **285** | 'GCCC in PS[1..19]' | **451** | 'PS1 forms bond' |
| **120** | 'GUU in PS[1..19]' | **286** | 'UAAA in PS[1..19]' | **452** | 'PS2 forms bond' |
| **121** | 'GUC in PS[1..19]' | **287** | 'UAAG in PS[1..19]' | **453** | 'PS3 forms bond' |
| **122** | 'GCA in PS[1..19]' | **288** | 'UAAU in PS[1..19]' | **454** | 'PS4 forms bond' |
| **123** | 'GCG in PS[1..19]' | **289** | 'UAAC in PS[1..19]' | **455** | 'PS5 forms bond' |
| **124** | 'GCU in PS[1..19]' | **290** | 'UAGA in PS[1..19]' | **456** | 'PS6 forms bond' |
| **125** | 'GCC in PS[1..19]' | **291** | 'UAGG in PS[1..19]' | **457** | 'PS7 forms bond' |
| **126** | 'UAA in PS[1..19]' | **292** | 'UAGU in PS[1..19]' | **458** | 'PS8 forms bond' |
| **127** | 'UAG in PS[1..19]' | **293** | 'UAGC in PS[1..19]' | **459** | 'PS9 forms bond' |
| **128** | 'UAU in PS[1..19]' | **294** | 'UAUA in PS[1..19]' | **460** | 'PS10 forms bond' |
| **129** | 'UAC in PS[1..19]' | **295** | 'UAUG in PS[1..19]' | **461** | 'PS11 forms bond' |
| **130** | 'UGA in PS[1..19]' | **296** | 'UAUU in PS[1..19]' | **462** | 'PS12 forms bond' |
| **131** | 'UGG in PS[1..19]' | **297** | 'UAUC in PS[1..19]' | **463** | 'PS13 forms bond' |
| **132** | 'UGU in PS[1..19]' | **298** | 'UACA in PS[1..19]' | **464** | 'PS14 forms bond' |
| **133** | 'UGC in PS[1..19]' | **299** | 'UACG in PS[1..19]' | **465** | 'PS15 forms bond' |
| **134** | 'UUA in PS[1..19]' | **300** | 'UACU in PS[1..19]' | **466** | 'PS16 forms bond' |
| **135** | 'UUG in PS[1..19]' | **301** | 'UACC in PS[1..19]' | **467** | 'PS17 forms bond' |
| **136** | 'UUU in PS[1..19]' | **302** | 'UGAA in PS[1..19]' | **468** | 'PS18 forms bond' |
| **137** | 'UUC in PS[1..19]' | **303** | 'UGAG in PS[1..19]' | **469** | 'PS19 forms bond' |
| **138** | 'UCA in PS[1..19]' | **304** | 'UGAU in PS[1..19]' | **470** | 'GC stretch of length >=4' |
| **139** | 'UCG in PS[1..19]' | **305** | 'UGAC in PS[1..19]' | **471** | 'G stretch of length >=3' |
| **140** | 'UCU in PS[1..19]' | **306** | 'UGGA in PS[1..19]' | **472** | 'AU stretch of length >=7' |
| **141** | 'UCC in PS[1..19]' | **307** | 'UGGG in PS[1..19]' | **473** | 'No. palindromes in PS[1..19]' |
| **142** | 'CAA in PS[1..19]' | **308** | 'UGGU in PS[1..19]' | **474** | 'Palindromes in PS[1..19]' |
| **143** | 'CAG in PS[1..19]' | **309** | 'UGGC in PS[1..19]' | **475** | Presence of repeats in PS[1..19]' |
| **144** | 'CAU in PS[1..19]' | **310** | 'UGUA in PS[1..19]' | **476** | GG(PS[1,2]) - GG(PS[18,19])' |
| **145** | 'CAC in PS[1..19]' | **311** | 'UGUG in PS[1..19]' | **477** | ‘GC content > 0.05’ |
| **146** | 'CGA in PS[1..19]' | **312** | 'UGUU in PS[1..19]' | **478** | ‘GC content > 0.1’ |
| **147** | 'CGG in PS[1..19]' | **313** | 'UGUC in PS[1..19]' | **479** | ‘GC content > 0.15’ |
| **148** | 'CGU in PS[1..19]' | **314** | 'UGCA in PS[1..19]' | **480** | ‘GC content > 0.2’ |
| **149** | 'CGC in PS[1..19]' | **315** | 'UGCG in PS[1..19]' | **481** | ‘GC content > 0.25’ |
| **150** | 'CUA in PS[1..19]' | **316** | 'UGCU in PS[1..19]' | **482** | ‘GC content > 0.3’ |
| **151** | 'CUG in PS[1..19]' | **317** | 'UGCC in PS[1..19]' | **483** | ‘GC content > 0.35’ |
| **152** | 'CUU in PS[1..19]' | **318** | 'UUAA in PS[1..19]' | **484** | ‘GC content > 0.4’ |
| **153** | 'CUC in PS[1..19]' | **319** | 'UUAG in PS[1..19]' | **485** | ‘GC content > 0.45’ |
| **154** | 'CCA in PS[1..19]' | **320** | 'UUAU in PS[1..19]' | **486** | ‘GC content < 0.95’ |
| **155** | 'CCG in PS[1..19]' | **321** | 'UUAC in PS[1..19]' | **487** | ‘GC content < 0.90’ |
| **156** | 'CCU in PS[1..19]' | **322** | 'UUGA in PS[1..19]' | **488** | ‘GC content < 0.85’ |
| **157** | 'CCC in PS[1..19]' | **323** | 'UUGG in PS[1..19]' | **489** | ‘GC content < 0.8’ |
| **158** | 'AAAA in PS[1..19]' | **324** | 'UUGU in PS[1..19]' | **490** | ‘GC content < 0.75’ |
| **159** | 'AAAG in PS[1..19]' | **325** | 'UUGC in PS[1..19]' | **491** | ‘GC content < 0.7’ |
| **160** | 'AAAU in PS[1..19]' | **326** | 'UUUA in PS[1..19]' | **492** | ‘GC content < 0.65’ |
| **161** | 'AAAC in PS[1..19]' | **327** | 'UUUG in PS[1..19]' | **493** | ‘GC content < 0.6’ |
| **162** | 'AAGA in PS[1..19]' | **328** | 'UUUU in PS[1..19]' | **494** | ‘GC content < 0.55’ |
| **163** | 'AAGG in PS[1..19]' | **329** | 'UUUC in PS[1..19]' | **495** | GG4^3)^(PS[1,2,3,4]) – GG4(PS[16,17,18,19])' |
| **164** | 'AAGU in PS[1..19]' | **330** | 'UUCA in PS[1..19]' | **496** | GG4(PS[1,2,3,4]) – GG(PS[18,19])' |
| **165** | 'AAGC in PS[1..19]' | **331** | 'UUCG in PS[1..19]' | **497** | GG(PS[1,2]) – GG4(PS[16,17,18,19])' |
| **166** | 'AAUA in PS[1..19]' | **332** | 'UUCU in PS[1..19]' |

1) PS denotes the position of nucleotides in the siRNA sequence.

2) GG denotes the thermodynamic stability of dinucleotides in siRNA antisense strand.

3) GG4 denotes the thermodynamic stability of tetranucleotides in siRNA antisense strand.
