# Supplementary material for: Reconsideration of In-Silico siRNA Design Based on Feature Selection: A Cross-Platform Data Integration Perspective
Source: PLoS One. 2012 May 24;7(5):e37879. doi: 10.1371/journal.pone.0037879 (PMC3360065; doi:10.1371/journal.pone.0037879)
Supplement: Table S2 — Sequence-specific study of the impact of the motif ‘UCU’. (DOC) [file pone.0037879.s002.doc]

### Table S2. Sequence-specific study of the impact of the motif ‘UCU’.

| **Starting nucleotide of motif** | **1** | **2** | **3** | **4** | **5** | **6** | **7** | **8** | **9** | **10** | **11** | **12** | **13** | **14** | **15** | **16** | **17** |
| --- | --- | --- | --- | --- | --- | --- | --- | --- | --- | --- | --- | --- | --- | --- | --- | --- | --- |
| **Dataset 1** | 67 | 71 | 54 | 58 | 55 | 57 | 53 | 56 | 56 | 61 | 58 | 52 | 70 | 61 | 50 | 55 | 67 |
| **Dataset 2** | 6 | 7 | 7 | 7 | 10 | 8 | 6 | 11 | 8 | 5 | 6 | 13 | 7 | 5 | 9 | 10 | 6 |
| **Dataset 3** | 7 | 7 | 7 | 7 | 7 | 7 | 7 | 7 | 7 | 7 | 7 | 7 | 7 | 7 | 7 | 7 | 7 |
| **Dataset 4** | 11 | 5 | 7 | 4 | 9 | 3 | 7 | 4 | 8 | 4 | 9 | 4 | 12 | 3 | 9 | 5 | 11 |
| **Dataset 5** | 2 | 2 | 2 | 3 | 3 | 2 | 3 | 3 | 3 | 3 | 4 | 5 | 3 | 2 | 2 | 2 | 2 |
| **Dataset 6** | 3 | 4 | 4 | 3 | 3 | 1 | 3 | 5 | 1 | 2 | 2 | 6 | 6 | 4 | 6 | 3 | 3 |
| **Dataset 7** | 0 | 0 | 0 | 0 | 2 | 0 | 0 | 0 | 0 | 0 | 0 | 1 | 0 | 1 | 0 | 0 | 0 |
| **Dataset 8** | 0 | 6 | 0 | 1 | 4 | 0 | 2 | 0 | 1 | 3 | 1 | 2 | 1 | 1 | 2 | 2 | 0 |
| **Dataset 9** | 3 | 2 | 0 | 1 | 0 | 0 | 3 | 2 | 0 | 1 | 1 | 1 | 3 | 1 | 2 | 1 | 3 |
| **Dataset 10** | 4 | 7 | 4 | 5 | 5 | 3 | 4 | 7 | 3 | 6 | 9 | 2 | 2 | 2 | 7 | 4 | 4 |
| **TOTAL (T1)** | 103 | 111 | 85 | 89 | 98 | 81 | 88 | 95 | 87 | 92 | 97 | 93 | 111 | 87 | 94 | 89 | 103 |

Analyzed are all entries of the respective dataset. Stated are the total numbers of sequences in each database that contain the motif at the nucleotide position indicated.
