# Supplementary material for: Reconsideration of In-Silico siRNA Design Based on Feature Selection: A Cross-Platform Data Integration Perspective
Source: PLoS One. 2012 May 24;7(5):e37879. doi: 10.1371/journal.pone.0037879 (PMC3360065; doi:10.1371/journal.pone.0037879)
Supplement: Table S3 — Sequence-specific study of the impact of the motif ‘UCU’. (DOC) [file pone.0037879.s003.doc]

### Table S3. Sequence-specific study of the impact of the motif ‘UCU’.

| **Starting nucleotide of motif** | **1** | **2** | **3** | **4** | **5** | **6** | **7** | **8** | **9** | **10** | **11** | **12** | **13** | **14** | **15** | **16** | **17** |
| --- | --- | --- | --- | --- | --- | --- | --- | --- | --- | --- | --- | --- | --- | --- | --- | --- | --- |
| **Dataset 1** | 50 | 47 | 32 | 30 | 37 | 27 | 35 | 35 | 34 | 30 | 26 | 37 | 38 | 32 | 32 | 31 | 50 |
| **Dataset 2** | 3 | 2 | 3 | 2 | 5 | 2 | 2 | 4 | 2 | 0 | 1 | 7 | 3 | 1 | 3 | 5 | 3 |
| **Dataset 3** | 3 | 6 | 3 | 4 | 4 | 2 | 3 | 4 | 4 | 4 | 5 | 4 | 5 | 5 | 1 | 2 | 3 |
| **Dataset 4** | 9 | 4 | 1 | 2 | 7 | 2 | 3 | 3 | 5 | 2 | 8 | 4 | 9 | 3 | 5 | 2 | 9 |
| **Dataset 5** | 2 | 2 | 1 | 3 | 3 | 2 | 3 | 3 | 3 | 3 | 4 | 4 | 3 | 2 | 0 | 2 | 2 |
| **Dataset 6** | 1 | 0 | 0 | 1 | 0 | 0 | 0 | 3 | 0 | 0 | 0 | 2 | 1 | 0 | 2 | 0 | 1 |
| **Dataset 7** | 0 | 0 | 0 | 0 | 1 | 0 | 0 | 0 | 0 | 0 | 0 | 1 | 0 | 1 | 0 | 0 | 0 |
| **Dataset 8** | 0 | 0 | 0 | 1 | 0 | 0 | 1 | 0 | 0 | 0 | 0 | 1 | 0 | 0 | 0 | 0 | 0 |
| **Dataset 9** | 3 | 2 | 0 | 0 | 0 | 0 | 2 | 1 | 0 | 0 | 1 | 1 | 2 | 0 | 2 | 1 | 3 |
| **Dataset 10** | 3 | 3 | 2 | 3 | 3 | 1 | 4 | 2 | 0 | 2 | 6 | 1 | 1 | 1 | 2 | 1 | 3 |
| **TOTAL (T2)** | 74 | 66 | 42 | 46 | 60 | 36 | 53 | 55 | 48 | 41 | 51 | 62 | 62 | 45 | 47 | 44 | 74 |
| $\frac{\text{T}2_{i}}{\text{T}1_{i}}\times\frac{\text{T}2_{i}}{\sum_{i=1}^{i=17} \text{T}2_{i}}$ | 0.059 | 0.043 | 0.023 | 0.026 | 0.040 | 0.017 | 0.035 | 0.035 | 0.029 | 0.020 | 0.030 | 0.046 | 0.038 | 0.026 | 0.026 | 0.024 | 0.059 |

Analyzed are only potent entries of the respective dataset. Stated are the total numbers of sequences in each database that contain the motif at the nucleotide position indicated.
