# Supplementary material for: Reconsideration of In-Silico siRNA Design Based on Feature Selection: A Cross-Platform Data Integration Perspective
Source: PLoS One. 2012 May 24;7(5):e37879. doi: 10.1371/journal.pone.0037879 (PMC3360065; doi:10.1371/journal.pone.0037879)
Supplement: Table S4 — Sequence-specific study of the impact of the motif ‘CCG’. (DOC) [file pone.0037879.s004.doc]

### Table S4. Sequence-specific study of the impact of the motif ‘CCG’.

| **Starting nucleotide of motif** | **1** | **2** | **3** | **4** | **5** | **6** | **7** | **8** | **9** | **10** | **11** | **12** | **13** | **14** | **15** | **16** | **17** |
| --- | --- | --- | --- | --- | --- | --- | --- | --- | --- | --- | --- | --- | --- | --- | --- | --- | --- |
| **Dataset 1** | 16 | 27 | 19 | 24 | 25 | 27 | 24 | 26 | 23 | 26 | 20 | 18 | 26 | 27 | 18 | 27 | 20 |
| **Dataset 2** | 4 | 8 | 4 | 4 | 8 | 5 | 8 | 5 | 4 | 6 | 8 | 7 | 8 | 6 | 4 | 6 | 9 |
| **Dataset 3** | 15 | 15 | 15 | 15 | 15 | 15 | 15 | 15 | 15 | 15 | 15 | 15 | 15 | 15 | 15 | 15 | 15 |
| **Dataset 4** | 2 | 5 | 1 | 5 | 1 | 6 | 1 | 6 | 1 | 5 | 3 | 6 | 2 | 6 | 2 | 6 | 2 |
| **Dataset 5** | 0 | 0 | 0 | 0 | 1 | 0 | 0 | 1 | 0 | 0 | 0 | 0 | 0 | 0 | 0 | 0 | 0 |
| **Dataset 6** | 0 | 0 | 0 | 1 | 0 | 1 | 0 | 0 | 1 | 0 | 1 | 0 | 3 | 0 | 0 | 0 | 0 |
| **Dataset 7** | 0 | 0 | 0 | 0 | 0 | 1 | 0 | 1 | 0 | 1 | 0 | 0 | 0 | 1 | 0 | 0 | 2 |
| **Dataset 8** | 2 | 1 | 2 | 0 | 1 | 1 | 0 | 0 | 1 | 1 | 1 | 1 | 1 | 2 | 0 | 3 | 1 |
| **Dataset 9** | 3 | 0 | 0 | 1 | 2 | 1 | 1 | 2 | 1 | 0 | 1 | 3 | 0 | 0 | 0 | 1 | 1 |
| **Dataset 10** | 2 | 2 | 4 | 1 | 0 | 4 | 4 | 6 | 1 | 4 | 1 | 6 | 4 | 4 | 1 | 2 | 3 |
| **TOTAL (T1)** | 44 | 58 | 45 | 51 | 53 | 61 | 53 | 62 | 47 | 58 | 50 | 56 | 59 | 61 | 40 | 60 | 53 |

Analyzed are all entries of the respective dataset. Stated are the total numbers of sequences in each database that contain the motif at the nucleotide position indicated.
