# Supplementary material for: Reconsideration of In-Silico siRNA Design Based on Feature Selection: A Cross-Platform Data Integration Perspective
Source: PLoS One. 2012 May 24;7(5):e37879. doi: 10.1371/journal.pone.0037879 (PMC3360065; doi:10.1371/journal.pone.0037879)
Supplement: Table S6 — Sequence-specific study of the impact of the motif ‘UCCG’. (DOC) [file pone.0037879.s006.doc]

### Table S6. Sequence-specific study of the impact of the motif ‘UCCG’.

| **Starting nucleotide of motif** | **1** | **2** | **3** | **4** | **5** | **6** | **7** | **8** | **9** | **10** | **11** | **12** | **13** | **14** | **15** | **16** | **17** |
| --- | --- | --- | --- | --- | --- | --- | --- | --- | --- | --- | --- | --- | --- | --- | --- | --- | --- |
| **Dataset 1** | 11 | 5 | 10 | 12 | 8 | 5 | 9 | 7 | 9 | 8 | 6 | 11 | 9 | 5 | 11 | 5 | 11 |
| **Dataset 2** | 4 | 1 | 0 | 0 | 1 | 1 | 1 | 1 | 2 | 1 | 1 | 1 | 0 | 0 | 1 | 1 | 4 |
| **Dataset 3** | 0 | 0 | 0 | 0 | 0 | 0 | 0 | 0 | 0 | 0 | 0 | 0 | 0 | 0 | 0 | 0 | 0 |
| **Dataset 4** | 5 | 0 | 5 | 0 | 6 | 0 | 6 | 0 | 5 | 0 | 5 | 0 | 5 | 1 | 5 | 1 | 5 |
| **Dataset 5** | 0 | 0 | 0 | 0 | 0 | 0 | 0 | 0 | 0 | 0 | 0 | 0 | 0 | 0 | 0 | 0 | 0 |
| **Dataset 6** | 0 | 0 | 0 | 0 | 1 | 0 | 0 | 1 | 0 | 1 | 0 | 1 | 0 | 0 | 0 | 0 | 0 |
| **Dataset 7** | 0 | 0 | 0 | 0 | 1 | 0 | 1 | 0 | 0 | 0 | 0 | 0 | 0 | 0 | 0 | 1 | 0 |
| **Dataset 8** | 0 | 1 | 0 | 1 | 0 | 0 | 0 | 0 | 0 | 0 | 0 | 0 | 2 | 0 | 0 | 1 | 0 |
| **Dataset 9** | 0 | 0 | 0 | 0 | 0 | 0 | 0 | 0 | 0 | 0 | 0 | 0 | 0 | 0 | 1 | 0 | 0 |
| **Dataset 10** | 1 | 1 | 0 | 0 | 2 | 1 | 3 | 0 | 1 | 0 | 1 | 2 | 0 | 0 | 0 | 2 | 1 |
| **TOTAL (T1)** | 21 | 8 | 15 | 13 | 19 | 7 | 20 | 9 | 17 | 10 | 13 | 15 | 16 | 6 | 18 | 11 | 21 |

Analyzed are all entries of the respective dataset. Stated are the total numbers of sequences in each database that contain the motif at the nucleotide position indicated.
