# Supplementary material for: Reconsideration of In-Silico siRNA Design Based on Feature Selection: A Cross-Platform Data Integration Perspective
Source: PLoS One. 2012 May 24;7(5):e37879. doi: 10.1371/journal.pone.0037879 (PMC3360065; doi:10.1371/journal.pone.0037879)
Supplement: Table S7 — Sequence-specific study of the impact of the motif ‘UCCG’. (DOC) [file pone.0037879.s007.doc]

### Table S7. Sequence-specific study of the impact of the motif ‘UCCG’.

| **Starting nucleotide of motif** | **1** | **2** | **3** | **4** | **5** | **6** | **7** | **8** | **9** | **10** | **11** | **12** | **13** | **14** | **15** | **16** | **17** |
| --- | --- | --- | --- | --- | --- | --- | --- | --- | --- | --- | --- | --- | --- | --- | --- | --- | --- |
| **Dataset 1** | 4 | 2 | 6 | 4 | 4 | 1 | 7 | 1 | 4 | 5 | 2 | 3 | 5 | 3 | 6 | 2 | 4 |
| **Dataset 2** | 2 | 1 | 0 | 0 | 0 | 1 | 0 | 1 | 0 | 0 | 0 | 1 | 0 | 0 | 1 | 0 | 2 |
| **Dataset 3** | 0 | 0 | 0 | 0 | 0 | 0 | 0 | 0 | 0 | 0 | 0 | 0 | 0 | 0 | 0 | 0 | 0 |
| **Dataset 4** | 4 | 0 | 5 | 0 | 2 | 0 | 4 | 0 | 2 | 0 | 1 | 0 | 4 | 1 | 4 | 1 | 4 |
| **Dataset 5** | 0 | 0 | 0 | 0 | 0 | 0 | 0 | 0 | 0 | 0 | 0 | 0 | 0 | 0 | 0 | 0 | 0 |
| **Dataset 6** | 0 | 0 | 0 | 0 | 0 | 0 | 0 | 0 | 0 | 0 | 0 | 0 | 0 | 0 | 0 | 0 | 0 |
| **Dataset 7** | 0 | 0 | 0 | 0 | 0 | 0 | 1 | 0 | 0 | 0 | 0 | 0 | 0 | 0 | 0 | 0 | 0 |
| **Dataset 8** | 0 | 0 | 0 | 0 | 0 | 0 | 0 | 0 | 0 | 0 | 0 | 0 | 0 | 0 | 0 | 0 | 0 |
| **Dataset 9** | 0 | 0 | 0 | 0 | 0 | 0 | 0 | 0 | 0 | 0 | 0 | 0 | 0 | 0 | 1 | 0 | 0 |
| **Dataset 10** | 0 | 0 | 0 | 0 | 2 | 0 | 2 | 0 | 0 | 0 | 0 | 1 | 0 | 0 | 0 | 1 | 0 |
| **TOTAL (T2)** | 10 | 3 | 11 | 4 | 8 | 2 | 14 | 2 | 6 | 5 | 3 | 5 | 9 | 4 | 12 | 4 | 10 |
| $\frac{\text{T}2_{i}}{\text{T}1_{i}}\times\frac{\text{T}2_{i}}{\sum_{i=1}^{i=16} \text{T}2_{i}}$ | 0.047 | 0.011 | 0.079 | 0.012 | 0.033 | 0.006 | 0.096 | 0.004 | 0.021 | 0.025 | 0.007 | 0.016 | 0.049 | 0.026 | 0.079 | 0.014 | 0.047 |

Analyzed are only potent entries of the respective dataset. Stated are the total numbers of sequences in each database that contain the motif at the nucleotide position indicated.
