# Supplementary material for: Reconsideration of In-Silico siRNA Design Based on Feature Selection: A Cross-Platform Data Integration Perspective
Source: PLoS One. 2012 May 24;7(5):e37879. doi: 10.1371/journal.pone.0037879 (PMC3360065; doi:10.1371/journal.pone.0037879)
Supplement: Table S8 — Sequence-specific study of the impact of the motif ‘CUU’. (DOC) [file pone.0037879.s008.doc]

### Table S8. Sequence-specific study of the impact of the motif ‘CUU’.

| **Starting nucleotide of motif** | **1** | **2** | **3** | **4** | **5** | **6** | **7** | **8** | **9** | **10** | **11** | **12** | **13** | **14** | **15** | **16** | **17** |
| --- | --- | --- | --- | --- | --- | --- | --- | --- | --- | --- | --- | --- | --- | --- | --- | --- | --- |
| **Dataset 1** | 47 | 63 | 60 | 60 | 50 | 55 | 44 | 59 | 41 | 50 | 57 | 52 | 51 | 49 | 56 | 50 | 50 |
| **Dataset 2** | 13 | 9 | 14 | 11 | 14 | 16 | 16 | 13 | 16 | 16 | 16 | 16 | 22 | 18 | 10 | 12 | 11 |
| **Dataset 3** | 20 | 20 | 20 | 20 | 19 | 19 | 19 | 20 | 20 | 20 | 20 | 20 | 20 | 20 | 20 | 20 | 20 |
| **Dataset 4** | 6 | 9 | 10 | 10 | 7 | 9 | 5 | 8 | 8 | 7 | 5 | 8 | 5 | 9 | 5 | 8 | 8 |
| **Dataset 5** | 1 | 2 | 2 | 1 | 3 | 2 | 2 | 2 | 3 | 3 | 3 | 3 | 4 | 3 | 2 | 4 | 2 |
| **Dataset 6** | 5 | 1 | 2 | 3 | 0 | 3 | 3 | 1 | 0 | 2 | 2 | 4 | 6 | 6 | 4 | 7 | 5 |
| **Dataset 7** | 0 | 0 | 0 | 0 | 0 | 0 | 0 | 0 | 0 | 0 | 0 | 0 | 0 | 0 | 0 | 0 | 0 |
| **Dataset 8** | 0 | 0 | 2 | 2 | 0 | 1 | 1 | 2 | 0 | 2 | 3 | 0 | 2 | 2 | 0 | 1 | 5 |
| **Dataset 9** | 0 | 3 | 3 | 0 | 1 | 1 | 2 | 1 | 2 | 0 | 4 | 1 | 3 | 1 | 1 | 1 | 1 |
| **Dataset 10** | 5 | 1 | 5 | 7 | 7 | 5 | 8 | 2 | 8 | 6 | 7 | 3 | 4 | 2 | 0 | 6 | 2 |
| **TOTAL (T1)** | 97 | 108 | 118 | 114 | 101 | 111 | 100 | 108 | 98 | 106 | 117 | 107 | 117 | 110 | 98 | 109 | 104 |

Analyzed are all entries of the respective dataset. Stated are the total numbers of sequences in each database that contain the motif at the nucleotide position indicated.
