# Supplementary material for: Reconsideration of In-Silico siRNA Design Based on Feature Selection: A Cross-Platform Data Integration Perspective
Source: PLoS One. 2012 May 24;7(5):e37879. doi: 10.1371/journal.pone.0037879 (PMC3360065; doi:10.1371/journal.pone.0037879)
Supplement: Table S10 — Sequence-specific study of the impact of the motif ‘GUU’. (DOC) [file pone.0037879.s010.doc]

### Table S10. Sequence-specific study of the impact of the motif ‘GUU’.

| **Starting nucleotide of motif** | **1** | **2** | **3** | **4** | **5** | **6** | **7** | **8** | **9** | **10** | **11** | **12** | **13** | **14** | **15** | **16** | **17** |
| --- | --- | --- | --- | --- | --- | --- | --- | --- | --- | --- | --- | --- | --- | --- | --- | --- | --- |
| **Dataset 1** | 43 | 33 | 40 | 42 | 39 | 27 | 38 | 45 | 39 | 31 | 52 | 43 | 36 | 32 | 29 | 30 | 43 |
| **Dataset 2** | 13 | 11 | 10 | 9 | 9 | 9 | 9 | 10 | 9 | 15 | 7 | 10 | 20 | 7 | 10 | 17 | 20 |
| **Dataset 3** | 13 | 13 | 13 | 13 | 13 | 13 | 13 | 13 | 13 | 13 | 13 | 13 | 13 | 13 | 13 | 13 | 13 |
| **Dataset 4** | 4 | 4 | 4 | 4 | 3 | 5 | 3 | 4 | 4 | 5 | 5 | 5 | 7 | 4 | 6 | 3 | 5 |
| **Dataset 5** | 0 | 1 | 0 | 1 | 0 | 0 | 0 | 0 | 0 | 1 | 1 | 0 | 0 | 0 | 0 | 0 | 0 |
| **Dataset 6** | 3 | 1 | 2 | 1 | 0 | 1 | 2 | 1 | 4 | 0 | 1 | 0 | 2 | 0 | 5 | 4 | 2 |
| **Dataset 7** | 0 | 0 | 0 | 0 | 0 | 0 | 0 | 0 | 0 | 0 | 0 | 1 | 0 | 0 | 1 | 0 | 0 |
| **Dataset 8** | 0 | 0 | 4 | 0 | 1 | 1 | 1 | 3 | 1 | 1 | 2 | 0 | 1 | 1 | 2 | 0 | 1 |
| **Dataset 9** | 1 | 0 | 0 | 0 | 1 | 0 | 1 | 2 | 1 | 1 | 0 | 2 | 2 | 2 | 1 | 0 | 1 |
| **Dataset 10** | 9 | 4 | 2 | 3 | 6 | 1 | 0 | 5 | 7 | 6 | 1 | 1 | 4 | 2 | 1 | 3 | 2 |
| **TOTAL (T1)** | 86 | 67 | 75 | 73 | 72 | 57 | 67 | 83 | 78 | 73 | 82 | 75 | 85 | 61 | 68 | 70 | 87 |

Analyzed are all entries of the respective dataset. Stated are the total numbers of sequences in each database that contain the motif at the nucleotide position indicated.
