# Supplementary material for: Reconsideration of In-Silico siRNA Design Based on Feature Selection: A Cross-Platform Data Integration Perspective
Source: PLoS One. 2012 May 24;7(5):e37879. doi: 10.1371/journal.pone.0037879 (PMC3360065; doi:10.1371/journal.pone.0037879)
Supplement: Table S12 — Sequence-specific study of the impact of the motif ‘GGG’. (DOC) [file pone.0037879.s012.doc]

### Table S12. Sequence-specific study of the impact of the motif ‘GGG’.

| **Starting nucleotide of motif** | **1** | **2** | **3** | **4** | **5** | **6** | **7** | **8** | **9** | **10** | **11** | **12** | **13** | **14** | **15** | **16** | **17** |
| --- | --- | --- | --- | --- | --- | --- | --- | --- | --- | --- | --- | --- | --- | --- | --- | --- | --- |
| **Dataset 1** | 54 | 23 | 32 | 33 | 36 | 35 | 26 | 33 | 35 | 38 | 38 | 30 | 31 | 33 | 36 | 33 | 30 |
| **Dataset 2** | 6 | 3 | 7 | 7 | 7 | 7 | 5 | 7 | 5 | 5 | 7 | 6 | 5 | 7 | 5 | 7 | 7 |
| **Dataset 3** | 22 | 17 | 17 | 17 | 17 | 17 | 17 | 17 | 17 | 17 | 17 | 17 | 17 | 17 | 17 | 17 | 17 |
| **Dataset 4** | 1 | 2 | 0 | 1 | 0 | 1 | 1 | 1 | 0 | 2 | 1 | 2 | 1 | 2 | 0 | 3 | 0 |
| **Dataset 5** | 0 | 1 | 0 | 0 | 0 | 0 | 0 | 0 | 1 | 0 | 0 | 1 | 0 | 0 | 0 | 0 | 1 |
| **Dataset 6** | 0 | 0 | 0 | 0 | 1 | 2 | 4 | 0 | 1 | 1 | 0 | 0 | 1 | 0 | 0 | 0 | 0 |
| **Dataset 7** | 0 | 0 | 1 | 0 | 0 | 0 | 0 | 0 | 0 | 1 | 0 | 1 | 0 | 0 | 0 | 0 | 0 |
| **Dataset 8** | 0 | 1 | 1 | 1 | 0 | 0 | 0 | 2 | 0 | 0 | 0 | 0 | 0 | 1 | 1 | 0 | 0 |
| **Dataset 9** | 3 | 1 | 2 | 2 | 0 | 2 | 1 | 1 | 0 | 2 | 0 | 1 | 1 | 1 | 0 | 0 | 1 |
| **Dataset 10** | 4 | 3 | 0 | 1 | 2 | 3 | 2 | 1 | 0 | 5 | 2 | 2 | 2 | 2 | 6 | 3 | 3 |
| **TOTAL (T1)** | 90 | 51 | 60 | 62 | 63 | 67 | 56 | 62 | 59 | 71 | 65 | 60 | 58 | 63 | 65 | 63 | 59 |

Analyzed are all entries of the respective dataset. Stated are the total numbers of sequences in each database that contain the motif at the nucleotide position indicated.
