# Supplementary material for: Reconsideration of In-Silico siRNA Design Based on Feature Selection: A Cross-Platform Data Integration Perspective
Source: PLoS One. 2012 May 24;7(5):e37879. doi: 10.1371/journal.pone.0037879 (PMC3360065; doi:10.1371/journal.pone.0037879)
Supplement: Table S13 — Sequence-specific study of the impact of the motif ‘GGG’. (DOC) [file pone.0037879.s013.doc]

### Table S13. Sequence-specific study of the impact of the motif ‘GGG’.

| **Starting nucleotide of motif** | **1** | **2** | **3** | **4** | **5** | **6** | **7** | **8** | **9** | **10** | **11** | **12** | **13** | **14** | **15** | **16** | **17** |
| --- | --- | --- | --- | --- | --- | --- | --- | --- | --- | --- | --- | --- | --- | --- | --- | --- | --- |
| **Dataset 1** | 4 | 13 | 17 | 12 | 14 | 19 | 10 | 15 | 16 | 15 | 12 | 7 | 10 | 12 | 10 | 10 | 13 |
| **Dataset 2** | 1 | 0 | 1 | 1 | 0 | 1 | 0 | 1 | 2 | 3 | 1 | 1 | 0 | 1 | 1 | 2 | 2 |
| **Dataset 3** | 3 | 1 | 3 | 0 | 1 | 2 | 3 | 1 | 5 | 5 | 3 | 2 | 0 | 3 | 3 | 2 | 5 |
| **Dataset 4** | 0 | 0 | 0 | 1 | 0 | 1 | 0 | 1 | 0 | 1 | 1 | 0 | 0 | 1 | 0 | 1 | 0 |
| **Dataset 5** | 0 | 1 | 0 | 0 | 0 | 0 | 0 | 0 | 1 | 0 | 0 | 1 | 0 | 0 | 0 | 0 | 1 |
| **Dataset 6** | 0 | 0 | 0 | 0 | 0 | 0 | 2 | 0 | 1 | 1 | 0 | 0 | 1 | 0 | 0 | 0 | 0 |
| **Dataset 7** | 0 | 0 | 0 | 0 | 0 | 0 | 0 | 0 | 0 | 1 | 0 | 0 | 0 | 0 | 0 | 0 | 0 |
| **Dataset 8** | 0 | 0 | 0 | 0 | 0 | 0 | 0 | 0 | 0 | 0 | 0 | 0 | 0 | 0 | 0 | 0 | 0 |
| **Dataset 9** | 0 | 0 | 0 | 0 | 0 | 0 | 0 | 0 | 0 | 2 | 0 | 0 | 0 | 1 | 0 | 0 | 0 |
| **Dataset 10** | 0 | 2 | 0 | 0 | 0 | 0 | 1 | 1 | 0 | 2 | 2 | 0 | 0 | 0 | 1 | 2 | 1 |
| **TOTAL (T2)** | 8 | 17 | 21 | 14 | 15 | 23 | 16 | 19 | 25 | 30 | 19 | 11 | 11 | 18 | 15 | 17 | 22 |
| $\frac{\text{T}2_{i}}{\text{T}1_{i}}\times\frac{\text{T}2_{i}}{\sum_{i=1}^{i=17} \text{T}2_{i}}$ | 0.002 | 0.019 | 0.024 | 0.011 | 0.012 | 0.026 | 0.015 | 0.020 | 0.035 | 0.042 | 0.018 | 0.007 | 0.007 | 0.017 | 0.011 | 0.015 | 0.002 |

Analyzed are only potent entries of the respective dataset. Stated are the total numbers of sequences in each database that contain the motif at the nucleotide position indicated.
