# Supplementary material for: Reconsideration of In-Silico siRNA Design Based on Feature Selection: A Cross-Platform Data Integration Perspective
Source: PLoS One. 2012 May 24;7(5):e37879. doi: 10.1371/journal.pone.0037879 (PMC3360065; doi:10.1371/journal.pone.0037879)
Supplement: Table S14 — Compositional feature ranking according to correlation coefficients ( R ). (DOC) [file pone.0037879.s014.doc]

### Table S14. Compositional feature ranking according to correlation coefficients (*R*).

| **Rank ID** | **Feature explanation** | **R** | **p-value** |
| --- | --- | --- | --- |
| **1** | 'U @ PS 1' | 0.2791 | 0.0003 |
| **2** | ‘GC content < 0.6’ | 0.2444 | 0.0170 |
| **3** | ‘GC content < 0.55’ | 0.2441 | 0.0038 |
| **4** | ‘GC content < 0.65’ | 0.2436 | 0.0043 |
| **5** | ‘GC content < 0.7’ | 0.2408 | 0.0024 |
| **6** | 'G @ PS 1' | -0.2137 | 0.0023 |
| **7** | 'C @ PS 1' | -0.1836 | 0.0075 |
| **8** | 'A @ PS 19' | -0.1443 | 0.1572 |
| **9** | 'A @ PS 10' | 0.1374 | 0.0288 |
| **10** | 'G @ PS 14' | -0.1366 | 0.0876 |
| **11** | 'A @ PS 1' | 0.1269 | 0.1147 |
| **12** | 'C @ PS 7' | -0.1264 | 0.0743 |
| **13** | 'G @ PS 13' | -0.1174 | 0.1282 |
| **14** | 'C @ PS 19' | 0.1120 | 0.2283 |
| **15** | 'A @ PS 18' | -0.0867 | 0.2145 |
| **16** | 'C @ PS 18' | 0.0705 | 0.2627 |
| **17** | 'G @ PS 19' | 0.0675 | 0.1340 |
| **18** | 'U @ PS 10' | 0.0064 | 0.4615 |
