# Supplementary material for: Reconsideration of In-Silico siRNA Design Based on Feature Selection: A Cross-Platform Data Integration Perspective
Source: PLoS One. 2012 May 24;7(5):e37879. doi: 10.1371/journal.pone.0037879 (PMC3360065; doi:10.1371/journal.pone.0037879)
Supplement: Table S15 — Motif feature ranking according to correlation coefficients ( R ). (DOC) [file pone.0037879.s015.doc]

### Table S15. Motif feature ranking according to correlation coefficients (*R*).

| **Rank ID** | **Feature explanation** | **R** | **p-value** |
| --- | --- | --- | --- |
| **1** | 'GGG in PS [1..19]' | -0.1561 | 0.0092 |
| **1** | 'G stretch of length >=3' | -0.1561 | 0.0092 |
| **3** | 'CUU in PS [1..19]' | 0.1420 | 0.0673 |
| **4** | 'UCU in PS [1..19]' | 0.1395 | 0.0562 |
| **5** | 'CCG in PS [1..19]' | -0.1352 | 0.1027 |
| **6** | 'GCC in PS [1..19]' | -0.1272 | 0.2189 |
| **7** | 'CU in PS [1..19]' | 0.1260 | 0.2946 |
| **8** | 'GGC in PS [1..19]' | -0.1259 | 0.1330 |
| **9** | 'CCC in PS [1..19]' | -0.0856 | 0.2423 |
| **10** | GUU in PS [1..19]' | 0.0362 | 0.3726 |
