# Supplementary material for: Reconsideration of In-Silico siRNA Design Based on Feature Selection: A Cross-Platform Data Integration Perspective
Source: PLoS One. 2012 May 24;7(5):e37879. doi: 10.1371/journal.pone.0037879 (PMC3360065; doi:10.1371/journal.pone.0037879)
Supplement: Table S16 — Thermodynamic feature ranking according to correlation coefficients ( R ). (DOC) [file pone.0037879.s016.doc]

### Table S16. Thermodynamic feature ranking according to correlation coefficients (*R*).

| **Rank ID** | **Feature explanation** | **R** | **p-value** |
| --- | --- | --- | --- |
| **1** | 'GG in PS [1,2]' | 0.3716 | 0.0000 |
| **1** | 'dG in NT[18,19]' | -0.1684 | 0.1044 |
